# Transformable nanoparticles to bypass biological barriers in cancer treatment

**DOI:** 10.1039/d2na00485b

**Published:** 2022-09-14

**Authors:** Mythili Ramachandran, Zhao Ma, Kai Lin, Cristabelle De Souza, Yuanpei Li

**Affiliations:** Department of Biochemistry and Molecular Medicine, University of California-Davis USA lypli@ucdavis.edu; Department of Medicinal Chemistry, Key Laboratory of Chemical Biology, School of Pharmaceutical Sciences, Cheeloo College of Medicine, Shandong University Jinan Shandong China; College of Food Science and Engineering, Ocean University of China Qingdao China; Department of Pathology and Stem Cell Research, Stanford University USA

## Abstract

Nanomedicine based drug delivery platforms provide an interesting avenue to explore for the future of cancer treatment. Here we discuss the barriers for drug delivery in cancer therapeutics and how nanomaterials have been designed to bypass these blockades through stimuli responsive transformation in the most recent update. Nanomaterials that address the challenges of each step provide a promising solution for new cancer therapeutics.

## Abstract 2

Nanomedicine provides a great strategy for emerging chemotherapeutics that can navigate the many biological barriers preventing effective drug delivery to tumors.

## Graphical abstract

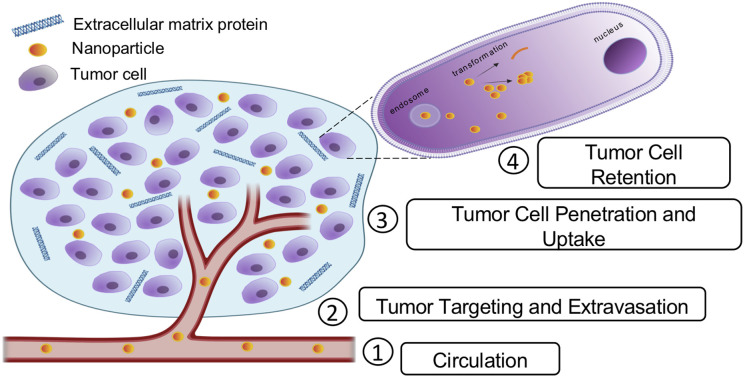

## Introduction

Cancer remains one of the leading causes of mortality despite significant advances in medicine and emerging treatments.^1^ Chemotherapy is the current preferred treatment for unresectable tumors and preventing metastasis. Many chemotherapeutic agents are small molecules that have systemic toxicity because of nonspecific interactions affecting healthy tissue, poor solubility and narrow therapeutic indices. To address these challenges, the use of nanomaterials is a method to improve existing chemotherapeutics with enhanced drug delivery to the tumor site along with imaging and detection of disease progression using a theranostic approach. Nanomedicine, referring to drugs approximately in the 1–100 nm range have the ability to be more easily functionalized, including their size, charge and surface properties, making them promising new generation chemotherapeutics.

**Fig. 1:**
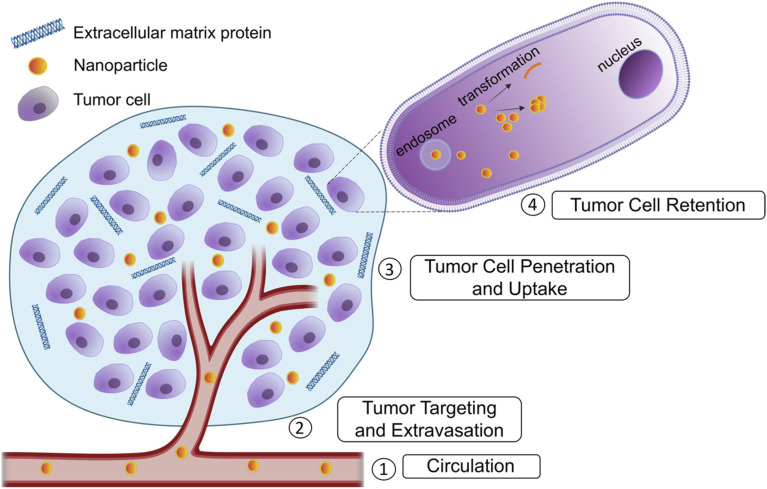
Biological barriers in drug delivery to tumors. Several barriers preclude drug delivery to tumors. These include severe destabilizing conditions during circulation, targeting the tumor location and extravasation into the tumor blood vessel. Next, the drug must penetrate the excess extracellular matrix (ECM) proteins and reach the tumor cells and finally, the drug must be retained in the tumor cells and avoid premature efflux. Made using Biorender.com.

Strategic design of nanoparticles to overcome each barrier for effective cancer treatment is necessary to improve current treatment options. The biological barriers include initial circulation in blood in which shear stress and interactions with blood proteins must be avoided. Next, drugs must target the tumor and avoid healthy cells, along with extravasating the blood vessel to reach the tumor tissue. The drug must then penetrate the dense extracellular matrix formed around tumors and enter the cell. Finally, the drug must remain in the cell long enough to exert an effect (Fig. 1). Nanomedicine provides many advantages to address these barriers.^2^ Initially, the enhanced permeability and retention (EPR) effect was the original explanation used to describe the superior capabilities of nanoparticles in tumor therapies. Nanoparticles preferentially enter the tumor due to the leakier vasculature surrounding the tumor and they are retained because of the lower lymphatic clearance.^2^ Recent reports have suggested additional mechanisms to explain nanoparticle uptake especially in desmoplastic tumors, namely transcytosis.^3,4^ Transcytosis involves the use of a receptor-mediated process to move the nanoparticle across the cell membrane. In the case of nab-paclitaxel, albumin bound paclitaxel facilitates transcytosis in the dense stroma of pancreatic cancer cells.^5,6^

Additionally, the size paradox that exists for nanoparticles needs to be accounted for when creating an optimal therapeutic agent. Smaller nanoparticles can easily traverse the bloodstream and be internalized by the tumor quickly. However, a smaller size results in quick clearance by the reticuloendothelial system (RES) and kidney.^2^ Larger nanoparticles avoid premature clearance and are retained for longer durations but cannot penetrate the tumor as efficiently.^7^ To overcome this paradox, transformability is a key method that could combine the advantages of both small and large nanoparticles to promote transcytosis in addition to the inherent EPR effect that makes nanoparticles attractive therapeutics.

Every transformable attribute should be unique and specific to a certain step in the cascade of drug delivery. The cascade has recently been coined CAPIR for circulation, accumulation, penetration, internalization, and release of the drug.^7^ The variation in tumor and patient type makes it very difficult to synthesize a nanoparticle that includes all the factors in the tumor uptake cascade without some form of stimuli responsive transformation at critical steps, as many favorable properties at one stage are adverse in another. Thus, the strategy of transformable nanoparticles holds promise for future advances in chemotherapeutics to tackle the barriers in cancer drug delivery.

## Blood circulation

Nanoparticle circulation is a pharmacokinetic barrier faced by all types of therapeutics.^8^ The shape and size of the nanoparticles largely dictate their chemical properties and their interactions in blood. Every nanoparticle and drug must have some degree of hydrophilicity to traverse the bloodstream. Nanoparticles also bind to plasma proteins and form a corona which can alter the size and properties of the drug.^8^ One such property is surface charge. Both positively charged nanoparticles and highly negatively-charged zeta potentials are cleared very quickly by the mononuclear phagocytic (MPS) system.^7,8^

The circulation time of the nanoparticle must be adjusted for each tumor type and case. For example, an extended circulation time is not necessarily a favorable factor if the nanoparticle cannot penetrate a tumor with poor biodistribution. In solid tumors, an excessively long plasma half-life could increase the potential of toxicity.^8^ However, in leukemia as well in delivering chemotherapies, this serves as an advantage to have a longer circulation time.^9,10^ To modulate the circulation time, the surface charge of the nanoparticle can be adjusted as previously mentioned. Several modification ligands and functional groups have been used for this purpose. Amines and imines are commonly used functional groups as their p*K*_a_s are near physiological conditions and they are subsequently protonated in more acidic environments. Zwitterionic ligands such as carboxy- or sulfobetaine have both positive and negatively charged groups which can be used to modify charge.^11^

Stability is a critical issue at this stage to prevent premature release of the encapsulated drug before the tumor site is reached. Our group and others have utilized a boronate ester cross-linkage between boronic acid and diols to improve nanoparticle stability until exposed to a lower pH in the target location.^12,13^ Another strategy for stability is the use of disulfide cross-linkages, such as in the core of micelles, which can withstand the shear stress in blood and improve circulation and delivery of the drug cargo.^14–16^ The cross-linking of micelles allows for a lower critical micelle concentration and prevents dissociation into unimers.

Stealth is another factor to include in the transformability of nanoparticles. To avoid immune clearance by the MPS there are a variety of methods. Most commonly a coating with polyethylene glycol (PEG)^17,18^ is used to avoid clearance with concurrent stabilization of the nanomaterial. After the success of Doxil, PEGylated doxorubicin (DOX), many materials have followed suit in using this polymer and its derivatives. For example, we have previously demonstrated that PEG_2000_ was used to create cross-linked nanoparticles that promote stability until their interaction with an acidic tumor microenvironment. PEGylation also provided surface charge modification. A Schiff base bond was used to PEGylate and cross-link the micelle and provide sensitivity to the acidic environment. The surface charge with PEG was much lower allowing for more stealth and stability in circulation until the tumor was reached in which the smaller, higher charged internal nanoparticles released from PEG could enter the tumor.^17^ In addition to PEG, many other hydrophilic polymers have been used to improve stability and evasion from the immune system such as PLGA [poly(lactic-co-glycolic acid)], POx [poly(2-oxazoline)] and other poly zwitterions.^8^

Alternatively, cell membranes can be used as a coating for nanomaterials to provide stealth in the blood.^18^ Several different types of cell membranes have been used to promote diverse functions. To avoid immune clearance, red blood cell (RBC) membranes are used to coat nanoparticles.^18–22^ Cell membranes are collected to form vesicles and loaded with the nanomaterial. Along with RBCs, platelets have also been used to avoid the MPS and they are easy to fractionize due to the lack of nuclei in both cell types.^17^ Additionally, nanoparticles can be camouflaged using exosomes, small extracellular vesicles that are secreted by cells.^23^ A recent study used exosomes from tumor cells exposed to DOX-loaded silicon nanoparticles as carriers allowing for better tumor accumulation and more extravasation from blood vessels.^24^

The protein corona effect is another phenomenon that nanoparticles face when in circulation. As the nanoparticle enters the bloodstream, plasma proteins accumulate on the surface and affect the zeta potential and nanoparticle size.^25^ One example, nab-paclitaxel, utilizes human serum albumin to facilitate entry of the drug into tumors, and many other examples have followed its lead in utilization of serum proteins. Zhang *et al.* utilized the protein corona properties of liposomes functionalized with a peptide that interacts with apolipoproteins.^26^ These liposomes exposed the receptor binding domain of the apolipoprotein and deliver DOX for glioma treatment.^26^ Hijacking the protein corona for delivery is a useful method to prevent the pitfalls of nanoparticle inactivation before cargo delivery.^25^

## Tumor targeting and extravasation

Targeting the tumor involves the use of ligands specific to tumor cells or tumor microenvironments. Many of the common targeting ligands have sensitivity to pH,^27^ enzyme reactivity,^25^ temperature,^28^ and other biomarkers.

**Fig. 2:**
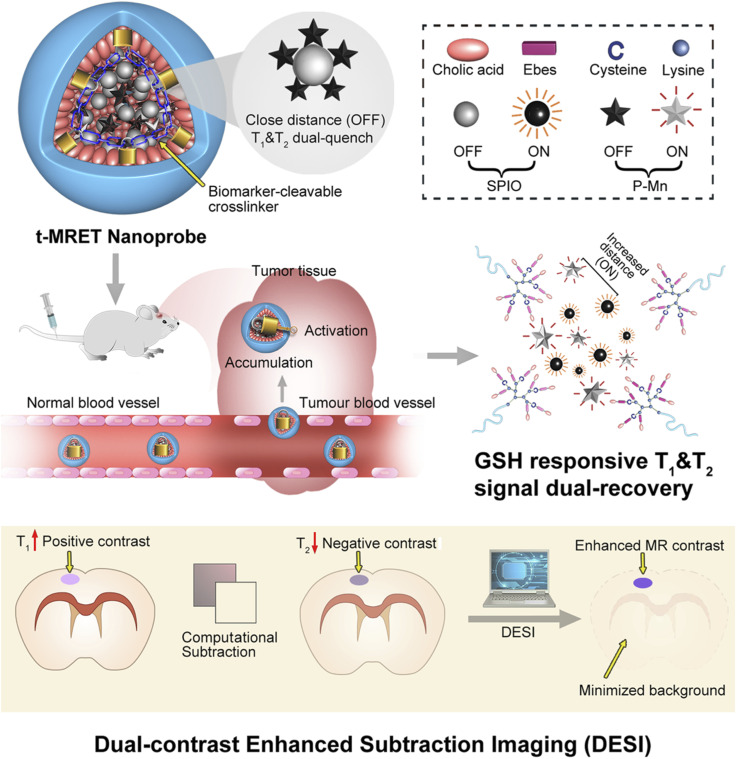
Schematic illustration of the TMRET nanotechnology and DESI utilizing GSH responsive transformation. Mn^2+^ conjugated to pheophorbide a serves as both an ‘enhancer’ in the T_1_ MRI signal and a ‘quencher’ in the T_2_ MRI signal, whereas the SPIO nanoparticle acts as an ‘enhancer’ in the T_2_ MRI signal and a ‘quencher’ in the T_1_ MRI signal. P–Mn and SPIO were coloaded into a disulfide crosslinked micelle to form TMRET nanoparticles. Upon interaction with reduced GSH (glutathione) the disulfide bond is cleaved allowing for signal measurement. Reproduced with permission from (ref. 29). © Springer Nature, 2020.

One such example of the nanotheranostic response to stimuli is the reaction to glutathione (GSH). In our group, we developed a probe for magnetic resonance imaging which was able to disassemble upon exposure to reduced glutathione, present in excessive amounts in the tumor (Fig. 2).^29^ Others have also utilized GSH for tumor targeting and transformation in treatment.^10,14,30^ Another condition that can be used for tumor targeting is the hypoxia often found in tumor microenvironments. Shen *et al.* utilized all-trans retinoic acid (ATRA) encapsulated in a nitroimidazole-modified hyaluronic acid-oxalate-camptothecin (CPT) conjugate to target the cancer stem cell niche in tumors. Under hypoxic conditions the ATRA is released causing differentiation of the cancer stem cells and exposing them to the CPT chemotherapeutic loaded in the nanoparticle.^31^

**Fig. 3:**
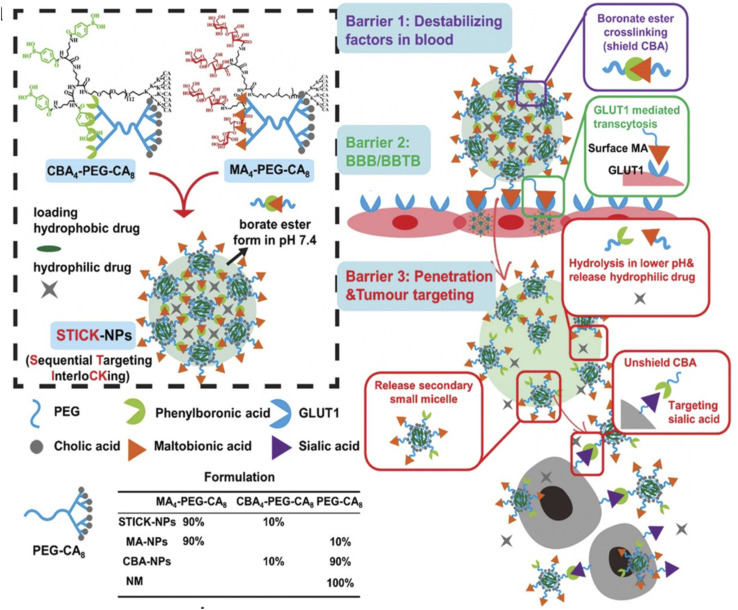
Design of transformable STICK-NPs and detailed multibarrier tackling mechanisms to brain tumors. The pair of targeting moieties selected to form sequential targeting in crosslinking (STICK) were maltobionic acid (MA), a glucose derivative, and carboxyphenylboronic acid (CBA), one type of boronic acid, and they were built into our well-characterized self-assembled micelle formulations (PEG–CA8). STICK-NPs were assembled by using a pair of MA4–PEG–CA8 and CBA4–PEG–CA8 with a molar ratio of 9 : 1 while intermicelle boronate crosslinkages, STICK, were formed between MA and CBA resulting in a larger nanoparticle size. Excess MA moieties were on the surface of the nanoparticles, while CBA moieties were first shielded inside the STICK to avoid nonspecific bindings. Hydrophobic drugs were loaded in the hydrophobic cores of secondary small micelles, while hydrophilic agents were trapped in the hydrophilic space between small micelles. In the following studies, we included several control micelle formulations including NM (no targeting), MA–NPs (single BBB targeting), and CBA–NPs (single sialic acid tumor targeting) nanoparticles (inserted table). In detail, STICK-NPs could overcome barrier 1 (destabilizing condition in the blood) by the intermicellar crosslinking strategy, barrier 2 (BBB/BBTB) by active GLUT1 mediated transcytosis through brain endothelial cells, and barrier 3 (penetration & tumor cell uptake) by transformation into secondary smaller micelles and reveal the secondary active targeting moiety (CBA) against sialic acid overexpressed on tumor cells in response to acidic extracellular pH in solid tumors. Reproduced with permission from (ref. 33). © Wiley (2020).

In brain tumors, an additional targeting barrier to bypass is the blood–brain-barrier (BBB). This barrier is composed of pericytes, astrocytes, and endothelial cells forming tight junctions which regulate the substances that can enter and exit the brain.^32^ In our lab, we have utilized a transcytosis method to navigate this barrier for brain tumor therapeutics. Two functional moieties were used to ensure BBB entry and transformation at the tumor site. First, maltobionic acid was used to facilitate GLUT1 transcytosis across the BBB and second, 4-carboxyphenylboronic acid was used to target sialic acid overexpressed in brain tumor cells (Fig. 3).^33^

## Tumor penetration and uptake

Regarding nanoparticle tumor penetration, the current consensus is that the EPR effect is responsible for the superior efficacy of nanoparticles for drug delivery to the tumor. The EPR effect cannot account for the excessive extracellular matrix (ECM) that surrounds many of the tumor types in which nanomaterials are effective.^24,34^ The overall pathway includes perfusion into the intratumoral vessels, extravasation and then penetration into the tumor mass.^36^ In the previous step, PEGylation provided the stealth needed for circulation. However, at this stage, entry into the tumor is limited by high PEG density.^35^ Transcytosis can be used at this step to facilitate tumor uptake by modifying materials with tumor targeting agents.

Within the tumor, high interstitial pressure is an added barrier preventing tumor penetration. To bypass this issue, many nanoparticles include anti-angiogenic factors to normalize the vasculature. Recently, Li *et al.* developed a stimuli responsive nanoparticle that utilizes thrombin loaded nanomaterials that also simultaneously deplete tumor-associated platelets to decrease abnormal angiogenesis.^36^ They showed nanomaterials composed of a poly(etherimide)-poly(lactic-*co*-glycolic acid)_2_ (PEI-PLGA_2_) copolymer for DOX encapsulation and an antibody R300 against platelets was functionalized on the surface through electrostatic interactions, allowing the final nanoparticle to release R300 and DOX at their targeted location to target platelets and tumor biomarkers.^36^ In addition to standard anti-angiogenic drugs, nitric oxide (NO) can be delivered, which can modulate angiogenesis and also promote vascular homeostasis and endothelial cell functions.^37^ Sung *et al.* made a NO delivery nanoparticle using a dinitrosyl iron complex [Fe(μ-SEt)_2_(NO)_4_] as the NO donor and poly-lactic glutamic acid (PLGA) as the method of controlled release of NO which showed efficacy in hepatocellular carcinoma models.^37^ Another method to decrease the higher interstitial pressure is to target the ECM proteins in the tumor microenvironment. The composition of the ECM is highly tumor dependent but generally includes collagen and fibrinogen.^7^ A collagen targeting material was made by Yao and colleagues in the form of a nanoenzyme capsule containing collagenase nanocapsules with heavy-chain ferritin nanocages containing DOX. Once the nanozyme capsule complex reached the mildly acidic tumor environment, the collagenase capsule was degraded, and collagenase could be activated to degrade collagen. The ferritin nanocage also promoted tumor penetration along with having tumor targeting ability. This nanoparticle also alleviated the hypoxic tumor environment with these components.^38^ Chen *et al .*recently made another hypoxia stimulus responsive material using a one-step method to combine paclitaxel, catalase, human serum albumin, and chlorine e6 into HAS-Ce6-Cat-PTX nanoparticles. These nanoparticles started at 100 nm for optimal blood circulation and upon reaching the tumor became small protein-drug complexes of 20 nm. Catalase responded to the endogenous H_2_O_2_ to form oxygen and alleviate the hypoxic environment.^39^

Size transformation is another strategy to improve tumor penetration and uptake. Many studies have utilized the properties of large to small transformation at the tumor site to prolong circulation as well as increase uptake at the tumor site. The main strategies include detachment of small vesicles from hybrid large-small nanoparticles or some degradation of the hybrid particle, a shell coating which detaches upon reaching the target location, and finally self-shrinkage.^40^ There are several different methods used to achieve these mechanisms. First, the use of pH will allow for transformation at a specific acidic tumor microenvironment. One ligand used was the amphiphilic copolymer PDPA_30_-*b*-PAMA_15_ which is hydrophobic in physiological pH and becomes hydrophilic in acidic pH resulting in a decrease in size from 35 to 10 nm.^41^ An even greater size decrease from 100 nm to 5 nm was seen with the use of 2,3-dimethylmaleicanhydride (DMA) which reacts in acidic conditions to amine groups and causes decomposition of a PCL-CDM-PAMAM/Pt system along with a charge reversal.^42^

**Fig. 4:**
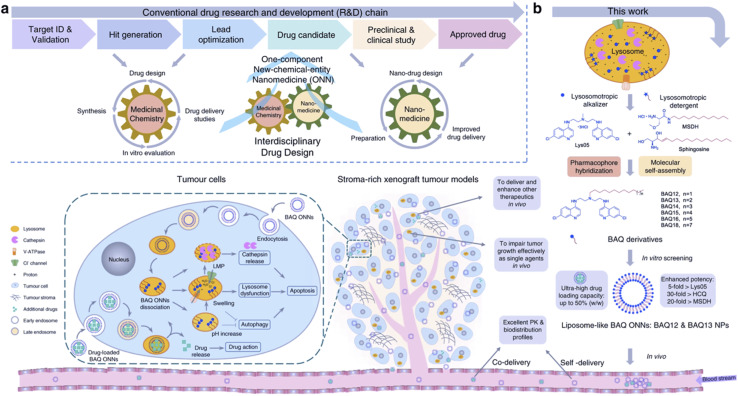
Tumor targeting with BAQ ONNs. (a) An interdisciplinary drug design strategy is proposed to integrate the conventional fields of medicinal chemistry and nanomedicine. Drugs are named as one-component new-chemical-entity nanomedicines (ONNs), which are designed according to the strategies of conventional drug design and molecular self-assembly so that they could acquire the advantages from the perspectives of both drug discovery and drug delivery. (b) The proof-of-concept experiment in this work: discovery of self-delivering lysosomotropic bisaminoquinoline (BAQ) derivatives for cancer therapy. The BAQ derivatives, generated from the hybridisation of lysosomotropic detergents and the BAQ-based autophagy inhibitor, can self-assemble into BAQ ONNs that show enhanced functions *in vitro*, excellent delivery profiles and significant *in vivo* therapeutic effects as single agents. Moreover, they also possess high drug-loading efficiency to deliver an additional drug into tumour sites, thus generating a promising application of combination therapy. Reproduced from (ref. 43) (CC BY 4.0).

Another facet of drug uptake includes uptake at the target location. Due to the size of many nanoparticles, uptake into the cell requires endocytosis resulting in subsequent trapping in the endosome and lysosome. One strategy to overcome this phenomenon is direct targeting of the lysosome. Lysosomal targeting is a viable strategy due to the dysregulation of lysosomal pathways when managing the higher metabolic requirements needed by tumor cells or to provide a method other than apoptosis for conventional chemotherapy-resistant cells. Our group has previously developed a lysosome-targeting small molecule that self assembles into a nanoparticle. Lysosome targeting caused disruption of its membrane combined with autophagy inhibition leading to potent efficacy in autophagy-dependent tumors (Fig. 4).^43^ Other nanomaterials developed ways to avoid endosomal trapping through three main mechanisms, cell penetrating peptides (CPPs), polymer-based nanomaterials that can create pores in the endosome, or lipid-based vehicles that can fuse with the endosome membrane to escape.^44^ Liposomes functionalized with an ionizable cationic lipid in their outer membrane can become protonated in the endosome and escape or disrupt the endosomal membrane.^45^ Some recent studies with CPPs include the development of a TAT analog conjugated with a methoxy PEG (MPEG)-poly(ε-caprolactone) (PCL) diblock copolymer which was unable to form a complex with DNA without TAT CPP mediation.^46^ CPPs often include short, positively charged peptide sequences that facilitate endosomal escape in response to a change in conformation upon reaching the lower pH endosome.

## Tumor retention

In tumor retention, the EPR effect is again called upon to describe the superior ability of nanoparticles to be retained in the tumor.^2^ The retention is seen through the limited lymphatic clearance present in tumors.

**Fig. 5:**
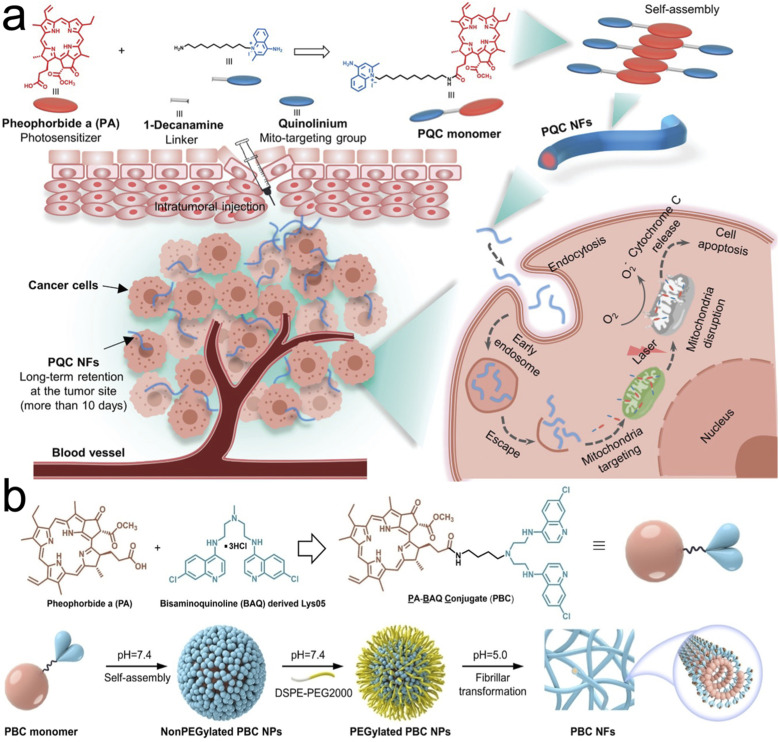
Tumor retention through transformation into nanofibers and nanofibrils. (a) Synthesis of single small molecule-assembled mitochondria targeting nanofibers (PQC NFs). The PQC monomer is a conjugate of PA and quinolinium. PQC NFs exhibited nanomolar cytotoxicity by mediating mitochondria-targeting phototherapy and they were retained long-term at the tumor site. With these advantages, PQC NFs achieved robust anticancer effects *in vivo* with a 100% complete cure rate after the administration of only a single dose. (b) Chemical structure of the PBC monomer containing pheophorbide a (PA) and bisaminoquinolone derived Lys05. Self-assembling and transformation behaviors of PBC at pH 7.4 and pH 5.0. PBC transformation into nanofibrils allowed for long term retention in the tumor site in oral cancer xenograft models. Reproduced with permission from (ref. 51 and 52). © Wiley (2020 and 2022).

Retention can be favored by transformation into various shapes apart from spherical vesicles that allow for decreased efflux of the drug. Nanoparticles in fiber conformations are more favorable for decreased tumor efflux while spherical particles are more favorable for circulation and tumor penetration^40^ as well as intravenous injection.^47^ Our group and others have developed transformable nanoparticles that prolong the drug retention at the tumor site by transformation into a nanofiber shape as a result of targeting receptor stimuli,^48–50^ or pH at the target location.^51,52^ Two such examples of nanofibers and nanofibril transformations are illustrated in Fig. 5. Other nanofibers using peptide-based self-assembly and transformation have been used in drug delivery to promote transformation at the tumor site which combine the benefits of tumor retention and biocompatibility.^48–50^ A recent study by Borkowska *et al.* showed accumulation of mixed charged nanoparticles that upon reaching the lysosome would aggregate into nanocrystals and be retained in the lysosome.^53^

Another method for tumor retention is to use small to large nanoparticle transformation. Aggregation of small nanoparticles at the site of the tumor prolongs the retention of the drug and improves efficacy. Additionally, nanoparticles can be optimized to include advantageous optical properties that allow for measurement of aggregation through emission or quenching of fluorescence^54,55^ or other measurable signals. Smaller size nanoparticles allow for easier tumor penetration but are also more likely to be cleared faster. For optimal tumor retention, aggregates of smaller particles can utilize the advantages of a smaller size without premature clearance. Stimulus response at the tumor site will allow for selective aggregation and prevent nonspecific interactions. Methods for this transformation include click reactions, electrostatic interactions, phase transitioning, swelling or self-assembling to transform into larger sizes and aggregation.^40^ One recent study by Hu *et al.* used a nanogel shell surface made of hyaluronic acid along with the enzyme, transglutaminase (TG). Upon tumor entry, TG catalyzed a peptide bond between lysine and glutamine allowing for 10 nm particles to aggregate into 120 nm in breast cancer models.^56^

Finally, tumor retention should be balanced with drug clearance to prevent systemic toxicity. An ideal nanoparticle should be retained long enough at the tumor site to exert its effect. A technique to ensure timely clearance particularly at nonspecific locations was used by Wang *et al.* where they entitled a tumor selective cascade activated self-retention system (TCASS). Their nanofibers underwent self-assembly at the tumor site while showing small molecule clearance in normal organs as the fibers became monomers.^57^

## Discussion/conclusion/perspective

The ability of nanomedicine to reach clinical translation requires more study into the pharmacokinetics and pharmacodynamics of nanomaterials. The heterogenous nature of tumors and patients is a challenge that should be addressed with adaptable nanomaterials specific to each case. New drug candidates along with improvement in preclinical models will allow for a more promising outlook for new chemotherapeutics. As seen in the discussion of these barriers, there are often contradictory conditions that promote increased efficacy at points of the delivery cascade. Additionally in the case of current personalized medicine, the patient and tumor type should be considered to provide maximum overall efficiency and most effective clinical translation. To summarize, nanoparticle blood circulation should be long enough to reach the target location while being concealed by the RES and MPS and should be stable enough to prevent premature drug release. Next, the nanoparticle should be able to penetrate the tumor and still be able to be internalized by the cell in the often-harsh tumor microenvironment. Finally, there should be tumor retention along with a timely clearance to prevent excessive toxicity. Stimulus-response allows nanoparticles to maximize effectiveness and provides the best outcome for a clinically relevant therapeutic. Novel treatments that account for tumor barriers and can adapt to tumor stimuli have the potential to revolutionize cancer therapy.

## Conflicts of interest

There are no conflicts of interest to declare.

## Supplementary Material
